# A framework to support dental radiography as part of diagnostic radiography practice

**DOI:** 10.4102/hsag.v31i0.3403

**Published:** 2026-07-06

**Authors:** Keshini Govindasami, Shenuka Singh

**Affiliations:** 1 Discipline of Public Health, School of Nursing and Public Health, College of Health Sciences, University of KwaZulu-Natal, Durban, South Africa; 2 Discipline of Dentistry, School of Health Sciences, University of KwaZulu-Natal, Durban, South Africa

**Keywords:** dental radiography, diagnostic radiographer, framework, participatory action research, precede-proceed model

## Abstract

**Background:**

The availability of evidence-informed frameworks to support learning interventions in dental radiography for diagnostic radiographers is limited. The framework used in this study was guided by participatory action research and adapted using the precede-proceed model. The framework is underpinned by social justice and systems and learning theory.

**Aim:**

This study aimed to determine and describe the application of a framework for supporting dental radiography as part of diagnostic radiography practice.

**Setting:**

Public and private radiology departments selected higher educational institutions from provinces of KwaZulu-Natal, Gauteng and Western Cape, inclusive of members from the Radiography and Clinical Technology Board at the Health Professions Council of South Africa.

**Methods:**

A sequential mixed-methods design was used for framework development, guided by the Precede-Proceed Model. Stage 1 involved a needs assessment, which comprised a quantitative Phase 1 and a qualitative Phase 2, which included an online questionnaire and semi-structured interviews, respectively. Stage 2 focused on the planning and delivery of the online Continuous Professional Development programme. Stage 3 involved a post-programme quantitative questionnaire to evaluate participant feedback and learning outcomes.

**Results:**

A strength of the framework is professional resilience and adaptability, enabling adaptation to technological advances and the changing needs of patients. The framework proposes a practical way to form targeted continuous professional development (CPD) initiatives, making it valuable in resource-constrained environments.

**Conclusion:**

The framework is beneficial for supporting dental radiography training and practices and promoting equitable access to dental radiography.

**Contribution:**

This framework adds value for supporting the practice of dental radiography for the diagnostic radiographers.

## Introduction

Programme planning and evaluation frameworks play a key role in guiding health planning by offering well-structured processes for planning, developing, implementing, monitoring and evaluating educational and clinical interventions (Gielen et al. 2022). Frameworks used in health programme planning provide structure, transparency, improve accuracy and strengthen stakeholder or community engagement (Fielding 2022). However, frameworks can become quite time-intensive, limit flexibility to emergent needs and require expertise (McMeekin et al. 2020). Despite these drawbacks, their benefits often outweigh their limitations, thereby making frameworks an essential mechanism in health programme planning (Fielding 2022).

There are essentially two key types of frameworks utilised in the health programme planning.

One common type is the logic model, which maps the sequence of inputs, activities, outputs, outcomes and long-term impacts of a health programme (Havsteen-Franklin, Cooper & Anas 2023).

Another widely utilised framework is the precede-proceed model, which has demonstrated its effectiveness in assisting health professionals in planning, designing and implementing health programmes (Ghaffari et al. 2021; Gielen et al. 2022).

The primary goal of the precede-proceed model is to ensure that all interventions are carefully planned for the benefit of the broader community, are evidence-based and can be effectively evaluated through their short- to medium-term and long-term goals (Crosby & Noar 2011).

The model looks to include the population of interest in developing the educational initiative by undertaking research to understand what their values, needs and interests are, then using this to structure a meaningful educational initiative that speaks to these issues (Ghaffari et al. 2021; Gielen et al. 2022). This model is aimed at ensuring that these educational initiatives are of value to the participants and their broader community (Gielen et al. 2022).

Despite the overall benefits of health programmes and planning frameworks, there remains a notable lack of evidence-informed frameworks to support learning interventions for diagnostic radiographers. Diagnostic radiography is a branch of radiography that fundamentally relates to diagnosing a patient’s condition through use of accurate and precise imaging techniques (Bushong 2021). This therefore requires diagnostic radiographers to possess core technical skills and competence that should be updated through lifelong learning (Makanjee, Zhang & Bergh 2023). Al Balushi, Watts and Akudjedu (2024) argued that although radiographers are motivated to engage in new learning, the structural mechanisms required to facilitate evidence-based learning, such as robust frameworks and institutional support, are either limited or underdeveloped. Similarly, Di Michele et al. (2024) called for more research into evidence-based practices to better guide and strengthen learning interventions within the diagnostic radiography profession.

This gap becomes even more evident within diagnostic radiography sub-areas, such as dental radiography, where programme planning frameworks are equally vital. High-quality dental imaging is dependent on the competence of the diagnostic radiographers and dental professionals, consisting of imaging techniques, patient positioning, appropriate selection of imaging techniques and accurate interpretation (Faculty of General Dental Practice [FGDP] (UK) & Public Health England 2020; International Atomic Energy Agency [IAEA] n.d.; Rai et al. 2018). Failure to apply these elements effectively can result in poor-quality images, which may compromise diagnosis, delay treatment and endanger patient safety (De-Azevedo-Vaz et al. 2013; Rai et al. 2018; Thakkar et al. 2019).

Despite these essential requirements, the available published literature suggests that there are gaps within training, exposure and ongoing professional development in dental radiography for diagnostic radiographers (Cheng et al. 2023; Ismail & Al Yafi 2024; Rai et al. 2018). This highlights the need for structured learning frameworks to support competence in the practice of dental radiography.

Furthermore, this misalignment between the expected competence and the availability of educational and training structures emphasises the limited use of structured evidence-based learning frameworks in dental radiography (Cheng et al. 2022, 2023; Rai et al. 2018). Therefore, enhancing such frameworks not only has the potential for supporting competence but also improving image quality, patient safety and overall diagnostic effectiveness (Faculty of General Dental Practice [FGDP] (UK) & Public Health England 2020; Siddique et al. 2024).

The purpose of this article is to illustrate how an adapted framework was used to systematically gather evidence to inform diagnostic radiography practice with specific reference to the provision for dental radiography services. This article forms part of a larger study that was conducted for degree purposes and is further explained next. This article, therefore, aimed to describe the framework used to guide the larger study, the underlying theoretical considerations and the methods used to show how the framework was operationalised. It is hoped that this evidence-based approach to review training and practice needs in diagnostic radiography can be replicated in other similar areas of health service provision.

## Brief description of the larger study

### Research methods and design

The following section provides a brief description of research methods and designs used for the larger study.

#### Research setting

In Phase 1, the questionnaire was distributed to qualified diagnostic radiographers in selected provinces, namely KwaZulu-Natal, Gauteng and the Western Cape. For Phase 2, semi-structured interviews were conducted with selected higher educational institutions from the provinces of KwaZulu-Natal, Gauteng and the Western Cape and also included members from the Radiography and Clinical Technology Board at the Health Professions Council of South Africa. Following this, Phase 3 involved a continuous professional development (CPD) invitation aimed at diagnostic radiographers from the same research setting identified in Phase 1 (Govindasami & Singh 2025a, 2025b, in press).

#### Design and sample

Phase 1 utilised a quantitative approach with a cross-sectional, correlational design, using both purposive and snowball sampling techniques (*n* = 207). Data were collected using an online questionnaire (Govindasami & Singh 2025a). Whereas Phase 2 included a qualitative methodology and incorporated an in-depth descriptive case study, using an exploratory and interpretivist design to conduct semi-structured interviews with purposefully selected academics (*n* = 8) and Radiography and Clinical Technology (RCT) board members (*n* = 4) (Govindasami & Singh 2025b). Finally, Phase 3 focused on the same population group as Phase 1 above and therefore, adopted the same research design.

### Data collection

Phase 1 included an online questionnaire that was sent out to 358 diagnostic radiographers to assess their knowledge, attitudes and practices (KAP); 207 responses were received, giving a response rate of 57.8%. Phase 2 consisted of semi-structured interviews, which initially targeted 20 academics; however, only eight academic staff consented to participate (Govindasami & Singh 2025a). Phase 2 also targeted five Radiography and Clinical Technology board members from the Health Profession Council of South Africa (HPCSA), of whom four consented to take part. This phase was conducted to explore curriculum, training and practice perspectives. Phase 2 also involved a document analysis of all publicly available curriculum documents to gain further insight into the problem statement (Govindasami & Singh 2025b).

Phase 3 involved planning, implementing and evaluating an online CPD programme that was designed for dental radiography and as recommended by study participants from the earlier phases (Govindasami & Singh in press). The study further employed data triangulation. The study first used an online questionnaire to establish baseline data and identify the key areas for further exploration (Govindasami & Singh 2025a).

This was followed by semi-structured interviews to gain a deeper insight into and validate findings from the questionnaire (Govindasami & Singh 2025b). In addition, all publicly available curriculum documents were analysed to compare the documented content with the study results and to further understand the reported inconsistencies (Govindasami & Singh 2025b). Phase 4 of the study synthesised findings from all phases to propose a framework to support and strengthen dental radiography training in SA.

### Data analysis

For Phase 1 and Phase 3, the quantitative component of the larger study data was analysed in conjunction with a statistician using Statistical Package for the Social Sciences. (SPSS) version 29.0. Descriptive statistics, including measures of central tendency, variability, frequency and goodness-of-fit tests, were used to summarise the data (Govindasami & Singh 2025a, in press). Phase 2, the qualitative component, utilised a coding expert, and data were triangulated and analysed using thematic and content analysis (Govindasami & Singh 2025b).

### Ethical considerations

Ethical clearance to conduct this study was obtained from the University of KwaZulu-Natal Humanities and Social Sciences Research Ethics Committee (No. HSSREC/00005832/2023). All procedures were conducted to ensure data privacy and protection. Gatekeepers’ permission and participants’ signed informed consent were obtained prior to conducting any data collection procedures.

## Results

Phase 1 of the larger study received a response rate of 57.8%. The online questionnaire items were centred around KAP. From the responses, 75.4% of participants agreed that they were aware of what dental radiography entails; 68.1% of participants reported not having adequate knowledge of the necessary dental radiography techniques; and 72.4% of participants did not know the key anatomical positioning landmarks in dental radiography. A total of 68.6% of participants disagreed that they were able to practise dental radiography techniques confidently and independently, while 87.5% of participants agreed that they were willing and keen to learn about dental radiography, and 89.9% of participants were open to gaining clinical experience in dental radiography (*p* < 0.001) (Govindasami & Singh 2025a).

Phase 2 reported key themes, which included the perceived understanding of the dental imaging scope of practice; under this theme, it was clear that dental radiography was part of the scope of practice and training for diagnostic radiographers. However, participants reported that there were inconsistencies in the depth of practice and variability across the training institutions. The second related theme was how limited exposure to undergraduate dental imaging training affects skills development. The limited availability of publicly accessible training documents creates an unclear picture of the extent to which dental imaging is incorporated into the undergraduate curriculum. Participants in this phase were in favour of more learning interventions (CPD) to be undertaken in this area (Govindasami & Singh 2025b).

Phase 3 reported that the post-programme assessment results (*n* = 54) showed that 96.3% of participants agreed that the CPD activity was useful and allowed them to learn something new. A total of 94.5% agreed that the CPD activity had met their expectations, and 92.5% agreed that the topic was relevant to their scope of practice. Additionally, 88.9% of participants agreed that the CPD activity assisted in improvement in their professional performance and competencies (*p* < 0.001) (Govindasami & Singh in press).

Phase 4 of the study synthesised findings from all phases to propose a framework to support and strengthen dental radiography training in SA. The framework development is further explained in the next section.

### Framework development

The proposed framework was adapted from the model developed by Green and Kreuter (2005), with modifications made to align it with the specific context and objectives of this study.

**FIGURE 1:**
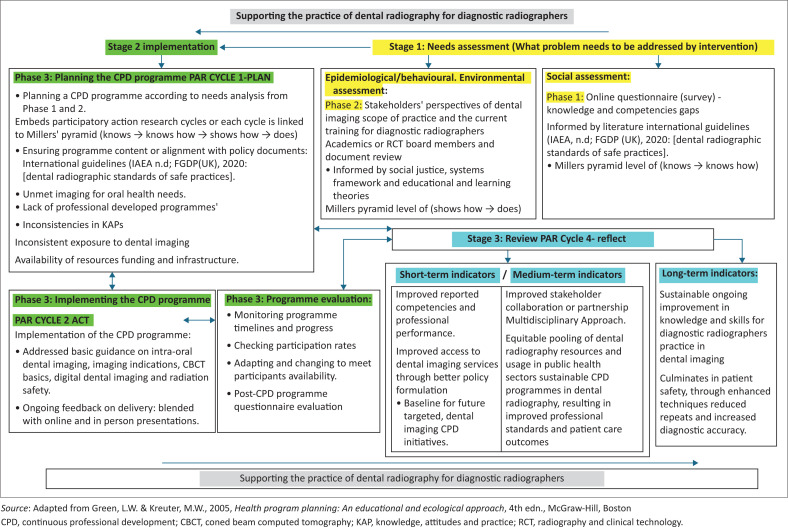
Proposed conceptual framework to support the practice of dental radiography for diagnostic radiographers.

The adapted framework used in the main study comprised three components, as illustrated in Figure 1, and is further described next.

#### Explaining the framework components and structure

These included: (1) Assessment stage, (2) implementation, and (3) review stage. The framework is underpinned by several theoretical considerations. This included the social justice theory (Rawls 1971), which looks at the framework through the lens of distributive justice of fair and equitable allocation of resources in dental imaging training; Millers’ pyramid (Miller 1990), which reflects on learning from (‘KNOW’ KNOW HOW’ ‘DOES’ and ‘SHOW’) in respect to dental imaging; and systems theory (Von Bertalanffy 1968), which further examines how the various subsystems influence the way dental radiography is taught and learnt.

The framework is also grounded in participatory action research (PAR) cycles (Feekery 2024; Upreti, Devkota & Maharjan 2024), thereby integrating some iterative components of PAR such as planning, action and reflection. Where Planning (PAR cycle 1) denotes the designing of the online CPD programme through a needs assessment, Action (PAR cycle 2) is the implementation of the online CPD programme and Reflection (PAR cycle 4) which deeply reflects on the sustainability of the learning intervention. These theoretical underpinnings guided the different highlighted components of the framework, as illustrated in Figure 1. The researcher also highlighted how the different phases of the study were included in these components.

***Needs assessment stage:*** Conducting a needs analysis ensured that the planned and learning intervention was relevant, evidence-based and responsive to the actual needs of a targeted group (Gielen et al. 2022). For Phase 1 of the study, and in keeping with the precede component, a needs assessment was conducted. This was achieved by means of an online questionnaire administered to diagnostic radiographers in selected provinces (Phase 1 of the study, *n* = 207) (Govindasami & Singh 2025a).

Participation in the study was voluntary, and after obtaining informed consent and ethical approval. The needs assessment also included semi-structured interviews with academics and RCT board members from HPCSA (Phase 2 of the study; *n* = 12) (Govindasami & Singh 2025b). The study’s findings indicated that there were reported knowledge and competence gaps in diagnostic radiographers’ technical and practical skills in dental imaging, thereby demonstrating social determinants for needs assessment (Govindasami & Singh 2025b).

***Implementation stage:*** For the implementation stage of the framework, the researcher incorporated the findings from the two phases above. The same population group and sample size as used in Phase 1 of this study were targeted and invited for the CPD programme. Sample size after estimated attrition was also considered, and the final sample size was *n* = 102. Essentially, the findings from Phases 1 and 2 suggested that there were shortcomings in participants’ knowledge and skills in dental radiography (Govindasami & Singh 2025a). The interviews with purposively selected academics and RCT board members further highlighted limited exposure to dental imaging, affecting skills development, and recommendations from stakeholders were to develop CPD initiatives in dental imaging (Govindasami & Singh 2025b).

Based on the data collected, the researcher proceeded to plan, develop and deliver the online CPD programme. The researcher chose to plan a CPD programme, as it allowed for tailored content to serve the needs (evidence-based) of the population rather than providing non-specific training. Participatory action research (Arshad et al. 2023; Feekery 2024) was embedded in this stage of the framework as it allowed for reflective learning (Adamson et al. 2023) and continuous practice change (Arshad et al. 2023; Upreti et al. 2024), allowing for the improvement of the CPD programme. The CPD programme included basic guidance on intra-oral dental imaging, imaging indications, coned beam computed tomography (CBCT) basics, digital dental imaging and radiation safety and was aligned to published literature on how to design learning activities for the health practitioner. The content topics were guided by the study findings and the literature for dental imaging (Faculty of General Dental Practice [FGDP] (UK) & Public Health England 2020; Goaz & White 1994; Manson-Hing 1990), used to train dental professionals in dental imaging. This topic content was guided in conjunction with dedicated dental academics who were involved in training dental professionals in dental imaging.

This stage required the researcher to do a process evaluation of the CPD programme to make sure that the planned learning intervention was delivered according to planned activities. The CPD combined online and face-to-face learning methods. This was combined with a post-programme questionnaire to obtain feedback on the delivered programme. Total post-programme questionnaires completed were (*n* = 54).

***Review stage:*** In the review stage of the framework, reflection allowed the researcher to reflect on the short-, medium-, and long-term indicators (Arshad et al. 2023; Feekery 2024). The process of reflection highlighted the CPD programme strengths and the need for continued support to further develop radiographers dental imaging skills. Short-term indicators targeted the immediate improvements for future learning intervention in dental imaging. Medium-term indicators highlighted strengthening interprofessional collaboration (IPC) and promotion of a multidisciplinary approach to align training and practice. Long-term indicators aimed to sustainably secure ongoing investments by professional bodies in learning interventions directed in dental radiography. It is advocated for sustainable learning initiatives, such as more practical stimulations needed in intra-oral imaging, access to digital stimulations and increased peer-led training, to improve image quality, reduce repeats and enhance patient safety (Parker et al. 2020).

Participatory action research (Arshad et al. 2023; Feekery 2024) provided a valuable mechanism to implement the online CPD programme. The concept of reflection was deeply relevant to the research processes, enabling the researchers to reflect at each stage of the research process. This allowed the researcher to make changes as and when needed to improve the overall delivery of the online CPD programme and to ensure that it met its stated objective, which was to develop a framework to support the practice of dental radiography for diagnostic radiographers in South Africa. In the process of reflection, the researcher was able to make recommendations for future CPD initiatives directed in dental radiography. Participatory action research (Arshad et al. 2023; Feekery 2024) follows a cyclical process of planning, acting, observing and reflecting, allowing for adjustments to be made as needed.

Following the description of the framework structure and components, it is equally important to understand the framework’s value and relevance and potential strengths.

## Discussion of the framework

The framework was developed to support the practice of dental radiography for diagnostic radiographers in South Africa. It focuses on keeping the knowledge and skills of diagnostic radiographers in the field of dental radiography updated through the promotion of continuous professional development initiatives. Cheng et al. (2023, 2025) also expressed strong agreement that a continuing education course in dental radiology improved participants’ knowledge, practical skills and attitudes, while stimulating greater motivation for continued learning.

The framework demonstrated the value of IPC in developing such targeted CPD initiatives in dental imaging (Delawala, Heymans & Christmal 2023). It further highlighted the need for more concerted stakeholder engagement required to address challenges in learning and practical exposure in the field of dental imaging. The framework focused on systems such as individual, social, behavioural, resource allocation, organisational practices and training institutions, which influence how dental radiography is taught and learnt.

This concept is supported by systems theory, which reviews how various components of a system interact or influence the way in which an individual may perform or behave (Von Bertalanffy 1968).

Furthermore, the framework provides an in-depth picture (Miller pyramid) of findings on what diagnostic radiographers may (KNOW; KNOW HOW) – (SHOW) as depicted in the findings from Phase 1, as compared to what happens in actual practice (DOES) as demonstrated in the findings from Phase 2 regarding dental imaging (Millers 1990). Therefore, the framework can be used to reflect diagnostic radiographers’ current competencies, practice and training in the field of dental radiography. Ultimately, this could be used to inform, plan or design future learning interventions directed to support the practice and learning of diagnostic radiographers in the field of dental radiography.

This study considered social justice theory to better inform findings that were derived from Phases 1 and 2.

This study provided a needs assessment of the social determinants and epidemiological data which assisted the framework to be evidence-based and better support the practice of dental radiography for diagnostic radiographers. The concept of distributive justice was considered as if students’ knowledge and skills were enhanced or given the opportunity to be exposed to dental imaging techniques, which would improve their understanding and practical application. Authors Kumsa et al. (2022) and Ganjitsuda (2025) state that adequate clinical placements and exposure to specific radiographic modalities are essential for learning and acquiring key competencies in the knowledge, skill and professional attributes required for clinical radiography. Furthermore, this in turn would facilitate improved access to dental imaging for all population groups as besides increasing the skilled work force in this domain. Patients would be given equal access to safe and high-quality care from skilled professionals who operate dental equipment.

Additionally, improving basic intra-oral dental imaging skills for diagnostic radiographers facilitates shared use of dental imaging resources, which in turn will increase access for citizens requiring oral healthcare diagnostic services. Distributive justice is focused on providing all citizens with an equal portion of resources and benefits (Rawls 1971). The framework provided a deeper understanding of how creating or encouraging stakeholders’ collaborative practices in generating more dental imaging CPD targeted initiatives could have the potential to ensure lifelong development of safe and effective practice (Faculty of General Dental Practice [FGDP] (UK) & Public Health England 2020; Makanjee et al. 2023). Thereby ensuring that all citizens have fair and equal exposure to similarly skilled professionals. Shared learning, also known as interprofessional education (IPE) and IPC, creates an understanding of professional roles and responsibilities among multidisciplinary teams (Delawala et al. 2023).

The next subsection highlights the main strengths and further applications of the proposed framework.

### Strengths of the framework

Looking at the framework from a systematic view demonstrates that it not only assists individual diagnostic radiographers but also has the potential to support learning across the wider dental radiography system in South Africa. It places emphasis on focused and targeted CPD initiatives to support continuous knowledge flow within the profession. It therefore creates feedback loops that enhance collective competence. By integrating mechanisms for sustainable learning, the framework contributed to a more adaptive and resilient professional system, one that can be capable of responding to dynamic technological advances and evolving patient needs.

A key strength of the framework is its clear step-by-step, evidence-based guideline for planning, designing and implementing dental radiography learning interventions. The framework provides practical processes for organising targeted CPD activities, thereby making it especially ideal for resource-constrained environments where access to this form of training is limited. The use of PAR principles (Arshad et al. 2023; Feekery 2024) of ‘plan, act, observe and reflect’ ensures that the framework remains flexible, responsive and adaptable to different contextual settings.

Another strength of the framework is its emphasis on reflective practices, which encouraged the researcher to critically examine knowledge and skills gaps, identify areas for improvement, and foster a deeper learning, better clinical judgement, and increase self-awareness (Aaron et al. 2021). Reflective practices also have the potential to foster adaptive expertise, which enables practitioners to respond to new technologies, procedural changes and complex patient situations (Adamson et al. 2023). Over time, this continuous cycle of reflection and application allows for sustained professional growth and improvement in patient outcomes and supports a culture of learning within healthcare teams (Aaron et al. 2021).

Finally, the methodological section reflecting the study’s mixed-methods design further strengthens the framework’s rigour and validity (McMeekin et al. 2020). The mixed-methods research approach further enabled the researcher to address the complex nature of the research questions, whereas data triangulation across study phases improved both the validity and reliability of the framework.

### Application of the proposed conceptual framework to other areas

The framework was developed to support the practice of dental radiography for diagnostic radiographers in South Africa, with core principles that can be applied across other domains of radiography and the broader health contexts. It is grounded in evidence-based practice and targets knowledge and skills gaps to promote competence-driven CPD and reflective learning.

Therefore, its components align with professional standards to encourage lifelong learning and strengthen healthcare quality.

The framework’s structured cycle of assessing learning needs, implementation of targeted CPD initiatives and evaluation of outcomes can therefore be effectively applied to areas such as CT, MRI and mammography. For example, it can guide CPD initiatives in skills development in dose optimisation and advanced imaging protocols in Computed Tomography (CT) and Magnetic Resonance Imaging (MRI) (Gallagher 2023; Mallinson, Hardy & Scally 2024) or in image quality and positioning in mammography (Seitzman, Pushkin & Berg 2021). It can be adapted to emerging fields, such as AI-assisted diagnostics and telemedicine, where ongoing ethical and technical competence is essential (Rainey et al. 2021).

Outside the radiography profession, the framework can inform CPD initiatives or stakeholders’ engagement in disciplines such as nursing, dentistry and allied health (Goldsberry 2018; Seaton et al. 2021). Overall, the framework supports evidence-informed practices, reflective learning and maintenance of professional competence required to deliver quality patient-centred care across healthcare settings.

### The limitations of the framework

There is a need for more innovative CPD methods or research to explore the feasibility and cost associated with such CPD learning interventions. This could include the development of further tools to gather such information, for example, focus group discussions and in-depth interviews. More effort could be put into the co-creation of research output through PAR. The design of the CPD learning activity required resources, such as access, funding, expertise and time that could become difficult to sustain, making careful planning essential. Furthermore, the framework is built on the assumption that there would be a high level of stakeholder engagement and willingness to collaborate among professional groups. This may not exist uniformly among institutions or different stakeholders and role players.

There may be resistance between the professional groups that may then hinder the framework recommendations to exposure and access. Lastly, the framework could encounter implementation barriers such as a lack of institutional, political, organisational culture and workforce support, which would require intensive planning and design phases to ensure successful implementation.

### Future recommendations and directions

Future stakeholder engagement or collaboration between diagnostic radiography and dentistry professionals should be encouraged to allow for alignment in training of dental radiography and increased exposure and access to dental equipment and resources. It is recommended that the basis of the systems theory model be utilised in resource allocation situations by identifying how low-cost strategies can improve access and training in dental radiography. It is further recommended to increase investments in institutional policy formulation and organisational cultural support to ensure effective implementation.

It is recommended that the framework application be tested between rural and urban areas to identify how different social or epidemiological factors may affect the application of the framework.

## Conclusion

The study offers a description of how a framework was applied through evidence-based research by first conducting a needs assessment, which then translated into practical outcomes that demonstrated immense potential for improving dental radiography training and practices for diagnostic radiographers. Overall, these efforts were grounded in the notion of equitable access to effective dental radiography services and, in turn, showed immense potential for improving access to dental radiography services for the diagnostic radiographer in oral health services in South Africa.
